# Quantifying the pharmacology of antimalarial drug combination therapy

**DOI:** 10.1038/srep32762

**Published:** 2016-09-08

**Authors:** Ian M. Hastings, Eva Maria Hodel, Katherine Kay

**Affiliations:** 1 Liverpool School of Tropical Medicine, Liverpool L3 5QA, United Kingdom

## Abstract

Most current antimalarial drugs are combinations of an artemisinin plus a ‘partner’ drug from another class, and are known as artemisinin-based combination therapies (ACTs). They are the frontline drugs in treating human malaria infections. They also have a public-health role as an essential component of recent, comprehensive scale-ups of malaria interventions and containment efforts conceived as part of longer term malaria elimination efforts. Recent reports that resistance has arisen to artemisinins has caused considerable concern. We investigate the likely impact of artemisinin resistance by quantifying the contribution artemisinins make to the overall therapeutic capacity of ACTs. We achieve this using a simple, easily understood, algebraic approach and by more sophisticated pharmacokinetic/pharmacodynamic analyses of drug action; the two approaches gave consistent results. Surprisingly, the artemisinin component typically makes a negligible contribution (≪0.0001%) to the therapeutic capacity of the most widely used ACTs and only starts to make a significant contribution to therapeutic outcome once resistance has started to evolve to the partner drugs. The main threat to antimalarial drug effectiveness and control comes from resistance evolving to the partner drugs. We therefore argue that public health policies be re-focussed to maximise the likely long-term effectiveness of the partner drugs.

Human malaria infections caused an estimated 214 million clinical cases and 438,000 deaths in 2015^1^. The relatively low case-fatality rate, even for the most virulent species, *P. falciparum,* is partly due to patient immunity acquired after repeated infections, but is also attributable to the timely provision of effective malaria drugs. There is a constant threat of malaria evolving resistance to available drugs and recent observations that resistance may have arisen to the most widely used antimalarial drug class, the artemisinins, has caused the World Health Organization to produce an emergency response in the Greater Mekong Sub-region to reduce its putative impact on the effectiveness of malaria treatment and control^2^. Artemisinin derivatives have to be deployed in combination with a ‘partner drug’ from a different drug class, the resulting drug combinations being known as artemisinin-based combination therapies (ACTs). One important operational question is to quantify the extent to which overall ACT cure rates may be threatened by resistance arising to their artemisinin components^3^. The impact of artemisinin resistance is generally assumed to be large (e.g. ref. 4 and 5) but there are few, if any, quantitative analyses to support this belief. Here we show that artemisinins make an extremely small contribution to overall ACT therapeutic capacity compared to their partner drugs, unless resistance has evolved to the partner drug, and argue that the debate over the impact and importance of artemisinin resistance needs to be re-interpreted in this light.

## Results

**Figure 1:**
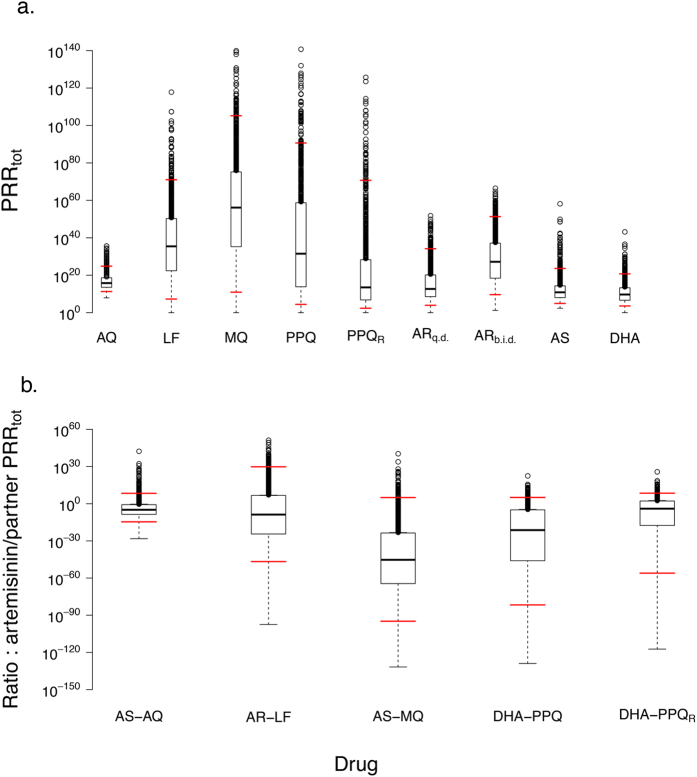
Boxplots of drugs’ therapeutic capacity quantified as PRR_tot_. (**a**) Individual drugs used in ACTs. (**b**) The contribution of artemisinin to overall ACT therapeutic capacity in a variety of ACTs; this is measured as the ratio artemisinin PRR_tot_: partner drug PRR_tot_. Note that in all plots the upper “whisker” of the boxplot lies immediately above the box and is difficult to distinguish. We identify the 5^th^ and 95^th^ centiles of the data by horizontal red lines. [The box delimits the second and third quartiles of the data (i.e. the inter-quartile range, IQR) with the horizontal line in that box representing the median value; the whiskers are the upper/lower quartile values plus/minus 1.5 times the IQR. Data points that lie outside the whiskers are regarded as outliers and are plotted individually. Note that the upper whiskers all lie virtually on top of the interquartile box due to the logarithmic scaling of the Y-axis]. Abbreviations: ACT = artemisinin combination therapy, AQ = amodiaquine, AR = artemether, AS = artesunate, DHA = dihydroartemisinin, LF = lumefantrine, MQ = mefloquine, PPQ = piperaquine, SP = sulfadoxine-pyrimethamine; Subscripts: b.i.d = twice daily dosing, R = resistance, q.d. = once daily dosing.

An intuitive, ‘simple’ approach, and a more sophisticated pharmacokinetic/pharmacodynamic (PKPD) modelling approach, can be used to quantify the therapeutic capacity of antimalarial drugs. This is most easily quantified as the total Parasite Reduction Ratio (PRR_tot_), of partner drugs and artemisinins used in the current generation of ACTs (see Methods section). The therapeutic capacities are given in Table 1. Partner drugs have far more therapeutic capacity than the artemisinins (Table 1) so the latter make only an extremely small contribution, typically ≪0.0001%, to overall therapeutic capacity of the ACT (Table 2). The PKPD method simulates 1,000 individual patients which allows the inter-patient variation in PRR_tot_ to be incorporated (Fig. 1a). These results show that the contribution of the artemisinins to total ACT therapeutic capacity is typically negligible when parasites are sensitive to the partner drug. The average contribution of artemisinins to ACTs based on the most widely used partner drugs (amodiaquine, lumefantrine, mefloquine, piperaquine) varies between 10^−10.5^ and 10^−30^ that of its partner drug using the simple method, and between 10^−9^ and 10^−46^ using the PKPD method. However, incorporating PK and PD variability suggests artemisinin may make a significant contribution in a small proportion of patients (Fig. 1b), although even if the artemisinin does make a significant contribution, the partner drug may still have sufficient therapeutic capacity to successfully eradicate the infection on its own. It is only when resistance has arisen to the partner drugs that artemisinins start to make a contribution to its ACT therapeutic capacity (Table 2). In summary, artemisinins make a negligible contribution to overall ACT therapeutic capacity when partner drugs are effective and only start to provide some protection once resistance starts to make the partner drug ineffective. Artemisinins play a role at this point (approximately halving failure rates^6^) but the long half-lives of partner drugs will further drive resistance eventually leaving artemisinins present as ineffective monotherapies^7^,^8^,^9^,^10^.

## Discussion

It is important to recognise the distinction between therapeutic capacity (the potential parasite killing) of a drug, and its actual killing capacity. In an idealised situation, where there is no resistance and both drugs are effective, it is clear that artemisinins contribute most to the short-term clearance of an infection. As a simple example, assume an infection has 10^11^ parasites, each artemisinin dose has a parasite reduction ratio over 48 hours (PRR_48_) of 10^4^, the partner drug has a PRR_48_ of 10^3^, then by 48 hours the artemisinin will have killed 10^8^ parasites and the partner drug 10^3^, so artemisinin will have killed >99.99% of the initial parasitaemia. It could be argued that this artemisinin killing is superfluous because the partner drug would have killed those parasites if the artemisinin had not been present, or was ineffective. However, it explains the basic principle that artemisinins are responsible for rapid initial parasite clearance, the high combined PRR_48_ observed after ACT treatment, and rapid alleviation of symptoms, while the partner therapeutic capacity is responsible for guaranteeing the long-term therapeutic outcome. The problems arise when this idealised situation does not apply and resistance starts to spread to one or both components of the ACT; this is the situation considered in this manuscript. Artemisinins in ACTs are largely protected by the partner drug providing there is no resistance to the latter: artemisinins have a short half-life so are always present with their partner drug (providing artemisinin monotherapy is not present) and the therapeutic capacity of the partner drug means there will be negligible selection for artemisinin resistance through ACT use (because parasites resistant to artemisinin would be killed by the partner drug and would have no selective advantage). Conversely, the partner drug has a long half-life so persists as a monotherapy in patients after the artemisinin has been eliminated; this long period of persistence throws substantial selection pressure on resistance to the partner drugs (see e.g. Fig. 1 of ref. 11). These dynamics means that ACT resistance is likely to arise in two distinct phases. Phase 1 is characterised by resistance eroding the therapeutic capacity of the partner drug to the point that its PRR_tot_ approaches that of the artesunate (Table 1, Fig. 1) and clinical failures start to occur to the ACT. This is supported by field evidence from South East Asia where a recent review^3^ noted that “a high ACT failure rate has only been observed where resistance to the partner drug is present, regardless of whether artemisinin resistance is present”. Phase 2 then starts because both the artemisinin and partner drugs have similar therapeutic capacities so both contribute to cure and hence selection pressure exists for resistance to each drug. The dynamics after this point are impossible to predict: the effectiveness of selection for resistance for each drug depends on the frequency of resistance mutations and the magnitude of their effect. Again, field data illustrates Phase 2 with both MQ- and PPQ-based ACTs showing failures attributable to both partner drug and artemisinin resistance^12^,^13^. An important practical consequence of these dynamics is that artemisinin ‘resistance’ will not encode cross-resistance to all ACTs. The therapeutic capacity, and outcome, is largely determined by the partner drug and it is only once the early stages of resistance have started to degrade the partners’ therapeutic capacity that any pre-existing resistance to the artemisinins may accelerate the final stages of overall ACT resistance.

The term “ACT” is often used as a synonym for “effective drug” and it is often not clear in any given context what impact is due to the drug specifically having an artemisinin component, or what impact followed simply because the ACT was an effective drug combination. For example, Bhatt and colleagues^14^ estimated that ACT provision contributed 22% to recent declines in the incidence of falciparum malaria in Africa. It is not clear whether this contribution is due to the properties of the artemisinin component itself or is attributable to effective partner drugs. The results presented above suggest the latter: the artemisinins will have made little impact on therapeutic outcome (by which we mean the eventual cure/failure of treatment), and the short half-life of the artemisinins compared to partner drugs means the former will not have contributed to post-treatment prophylaxis of the drug. If artemisinins contribute little to overall ACT therapeutic capacity, and hence to therapeutic outcome, the obvious question is what do they contribute? One important property is their rapid action which alleviates symptoms, and may prevent patients progressing from uncomplicated to complicated or severe malaria during the first 24 hours post-treatment. A second factor is that artemisinins kill immature gametocytes during the early stages of their ~10-day maturation period. Gametocytes have no clinical impact so this activity against immature gametocytes has no clinical implications. However, it may have a public health benefit in reducing onward transmission of malaria. Recent modelling work investigating the impact of adding primaquine (which kills mature gametocytes) to ACTs suggested its impact was negligible (e.g. ref. 15). If primaquine’s effect on killing mature, infectious gametocytes was negligible in terms of public-health, it seem logical to suppose that artemisinin’s ability to kill immature, non-infectious gametocytes will have a similarly small impact. This point is important because many of the commentaries on the threat posed by artemisinin resistance stress the public-health impact, for example that artemisinin resistance is “a major threat to further advances in malaria control”^4^, that it “threatens worldwide initiatives to control and eliminate malaria”^16^, or that “the prospects for the elimination of malaria, are now threatened by the emergence of artemisinin resistance”^17^. We argue that it is important to place artemisinins within the context of ACT action. In particular, it is essential to distinguish their impact on rapidly reducing parasite load (at which they excel), from their ability to contribute to eventual therapeutic outcome, i.e. cure (which is often marginal; see Table 2 and Fig. 1). Based on our analyses, and more general properties of ACTs, there are, in our opinion, five major implications for resistance, existing or potential, to the current generation of ACTs.

First, the threat posed by resistance evolving to the partner drug. In an ACT where no resistance is present to either drug, the partner drug typically contributes >99.9999% of the therapeutic capacity (Table 2, Fig. 1b) and is mainly responsible for ensuring the successful therapeutic outcome of treatment. Their long half-lives mean they persist as vulnerable monotherapies for significant periods of time after the short half-life artemisinin have been eliminated. These periods constitute a “window of selection” for resistance^10^,^18^ which is one of the three key drivers of resistance^7^. Their actions can be detected in the field (e.g. ref. 19 and 20) and can potentially shorten the useful therapeutic lifespan of ACTs (e.g. Box 2 of reference 8) irrespective of whether or not resistance is present to the artemisinin component.

Second, it is doubtful whether administering artemisinins once-daily is the optimal regimen given their very short half-lives. Twice-daily dosing appears to be a much more efficient use of artemisinins (see artemether in Table 1) and is discussed in more detail elsewhere^21^. This strategy of twice-daily dosing may therefore restore falling artemisinin effectiveness and also reduce the ability of artemisinin resistance to spread through parasite populations. In particular, the use of probably sub-optimal (i.e. once daily dosing) artemisinin regimens in efforts to eradicate putative artemisinin-resistant malaria populations (e.g. ref. 2) seems, at least to us, contra-indicated. This can be most conveniently achieved by simply splitting the ACT daily dosage into two halves, including the partner drug dosage to avoid having to provide artemisinin monotherapies.

Third equally, we share the widespread concerns about artemisinin resistance as detected by decreased parasite clearance times (e.g. refs 4 and 5). Currently, this appears to be the main focus in the literature but it is clear that the concerns need to spread much wider than simply focussing on artemisinin “resistance”. Resistance may compromise the effectiveness of artemisinins in the monotherapy of severely ill patients, but there appears to be less cause for alarm about in their role in ACTs. We do not share the cataclysmic predictions of its public-health impact claimed by some authors (see above) but we are likely to lose the extension of the therapeutic life-span that artemisinin can provide once partner drugs start to fail (Table 2B), which would allow time for policy changes to be implemented.

Third equally, it is possible that increased resistance to partner drugs and artemisinins is already present but has remained undetected through overreliance on parasite clearance rates as surveillance tools. Immunity is known to make a large contribution to parasite clearance rates^22^,^23^ and simulations suggest immunity completely dominates the clearance dynamics of parasites following artemisinin treatment unless drug effectiveness falls to very low levels (<~10% of original killing)^24^. The impact of human immunity in clearing erythrocytes containing dead or dying parasites makes parasite clearance rates highly insensitive and non-specific diagnostics of resistance^24^,^25^,^26^,^27^. Consequently, parasite clearance rates represent poor surveillance tools and even large increases in drug resistance (to both the artemisinins and the partner drugs) may already be present in populations but remain undetected.

Fifth, the use of clearance rates as metrics of ACT resistance and effectiveness may miss substantial increases in ACT effectiveness that could be obtained by changes in deployment regimen. For example, Guinea Bissau overcame chloroquine resistance by the simple (but potentially toxic) strategy of doubling the dosage given^28^. Increasing dosage is one of the easiest ways to overcome resistance and it is highly likely that all antimalarial drugs were initially deployed at too low a dose; most have had their dosage increased^29^. The “problem” with ACTs is that failure rates are currently low (but see refs 11,12 and 30) so drug effectiveness cannot be directly assessed by clinical trials using cure/failure rates as the end follow-up. That leaves pharmacological modelling as the main (and possibly the only) way to quantify the impact of regimen changes for example, the proposed move towards triple-drug combination therapies for malaria^31^,^32^.

Modelling plays an increasingly important role in planning malaria control and interventions^33^ and requires a component that quantifies the impact of drugs on treatment outcome; for example Slater and colleagues^34^ recently modelled the public-health impact of artemisinin and partner drug resistance. Our recent pharmacological modelling work on ACTs (op cit) has enabled us to contribute to this modelling agenda, and wider debates, by placing concerns about artemisinin resistance in a more objective, quantitative framework with ramifications for both treatment and public-health applications.

## Methods

The simplest way to quantify the therapeutic capacity of antimalarial drugs is through their parasite reduction ratio (PRR)^35^,^36^, a strategy that dates back to Sir Ronald Ross^37^ who calculated a drug’s “single-dose reduction rate”. The PRR is defined as the ratio of the number of parasites at time of treatment divided by the number after a given amount of time has elapsed post-treatment. This time period is normally 48 hours as this is the time taken for *P. falciparum* parasites to pass through their asexual erythrocytic life cycle. We denote this metric as PRR_48_, so if a drug has a PRR_48_ of 10^3^ it indicates that a proportion of 10^−3^ parasites survive one erythrocytic cycle in the presence of the drug. If a drug is present at active concentrations (i.e. killing at a maximum rate; see Supplementary Information File for further details) for *c* erythrocytic cycles post-treatment, then its therapeutic capacity can be quantified as the total PRR it has accumulated over those cycles, i.e.




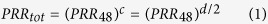




Note that *c* is most conveniently obtained by estimating the number of days, *d*, post treatment that a drug is active, and dividing it by 2 to obtain the number of 48-hour erythrocyte cycles. So for example, if a drug is actively killing parasites for d = 20 days after treatment, it is killing for c = 20/2, i.e. ten 48-hour life cycles. A PRR_48_ of 10^3^, typical of partner drugs^35^,^36^, would therefore generate a PRR_tot_ = (10^3^)^10^ = 10^30^, thus implying that only 10^−30^ parasites present at the start of treatment would survive the 20 days of active parasite killing. Given that malaria infections rarely exceed around 10^12^ parasites, any drug with a value of PRR_tot_ > 10^13^ implies a fully effective drug but PRR_tot_ serves as a key theoretical metric for the therapeutic capacity of the drug. As an example, lumefantrine has a PRR_tot_ of ~10^35^ (Table 1). It will never be required to remove 10^35^ parasites from a single infection but this metric of therapeutic capacity gives an indication of how much ‘margin of error’ is associated with the drug therapy and hence how robust it is to variation in patients’ pharmacokinetics (e.g. Fig. 1a), their adherence to the regime, existing variation in parasites drug susceptibility, and to the first stages of drug resistance.

Artemisinins persist at active concentrations for much shorter periods of time, *d*, post-treatment but are generally ascribed a PRR_48_ = 10^4^ (refs 35 and 36). It is not clear whether this PRR_48_ occurs after a single dose or after multiple doses but here we make the assumption (generous to artemisinins) that it occurs after a single dose. Three once-daily doses of an artemisinin, as occurs in most ACT regimens, will therefore generate




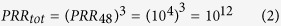




One exception to this ACT regimen of once-daily dosing over three days is the combination of artemether with lumefantrine which is given twice-daily over three days. When the recommended daily dose of artemether (~4 mg/kg) is given as one daily dose, artemether persists for around 6 hours post-treatment. When the same dose is halved (~2 mg/kg) and given twice daily, its duration of persistence post-treatment is reduced by one elimination half-life (around 40 minutes to one hour) so the period of active parasite killing following each of the six doses is reduced to approximately 5 hours per dose. This gives PRR_tot_ for twice daily artemether as




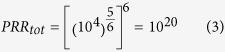




This increase in artemether therapeutic capacity (*cf* Equation 2) is caused solely by it being dosed twice-daily while maintaining the same daily total dose. It would apply to all artemisinins dosed twice-daily and is a remarkable result that suggests that artemisinins regimens given once-daily are sub-optimal (discussed more fully elsewhere^21^).

This “simple” method to assess drug effectiveness requires knowledge only of clinical observations already widely cited and accepted in the literature (i.e. PRR_48_ and drug persistence post-treatment; see Supplementary Information File for details) and familiarity with the simple algebraic rule that (10^x^)^y^ = 10^xy^ used to produce Equation 1 and Equation 2. We also use a more nuanced PKPD approach to quantify a more sophisticated estimate of the contribution of artemisinins to overall ACT drug killing. The PKPD methodology recognises that pharmacological parameters vary enormously between patients depending on how they absorb, metabolise, distribute and eliminate drugs (their pharmacokinetics, PK) and between malaria parasites depending on their drug sensitivity (their pharmacodynamics, PD) so artemisinins may play a more significant role in treatment of some patients, e.g. those who rapidly eliminate the partner drug and/or whose parasites are naturally less sensitive to the partner drug. The basis of the PKPD method is the following equation




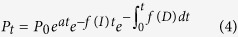




which may be easily understood intuitively: it states that the number of parasites, *P*_*t*_, present at time *t* after treatment depends on the initial number of parasites present at time of treatment, *P*_0_, augmented by growth, *a,* that has occurred during time *t*, offset against the amount of immune killing over time *t*, −*f*(*I*)*t* and also offset against the amount of drug killing over time *t*, i.e. *f*(*D*). Immunity is generally ignored^38^ so *f*(*I*) = 0. This is a fairly standard method for investigating the treatment of infectious diseases (for a review, see ref. 39). It was first applied to malaria by Hoshen *et al.*^40^ and Austin *et al.*^41^, further developed by Hoshen and colleagues^42^,^43^,^44^,^45^, sporadically used subsequently by other authors (e.g. refs 18,46 and 47) and more recently taken up by ourselves to develop the methodological extensions and calibrations required to model ACT treatment^21^,^24^,^38^,^48^,^49^,^50^,^51^,^52^. Equation 4 can be solved to find the predicted minimum number of parasites post-treatment, *P*_min_ which allows PRR_tot_ to be calculated as *P*_0_/*P*_min_.

## Additional Information

**How to cite this article**: Hastings, I. M. *et al.* Quantifying the pharmacology of antimalarial drug combination therapy. *Sci. Rep.* **6**, 32762; doi: 10.1038/srep32762 (2016).

## Supplementary Material

Supplementary Information

## Figures and Tables

**Table 1:** The therapeutic capacity of a range of antimalarial drugs, quantified by their PRR_tot_.

Drug	PRR_tot_(simple method)	PRR_tot_ (PKPD method)	Monotherapy cure rate (PKPD method)
*Partner drugs (sensitive):*			
AQ	(10^3^)^15/2^ = 10^22.5^	1.7 × 10^20^	86%
LF	(10^3^)^24.5/2^ ≈ 10^37^	2.7 × 10^35^	90%
MQ	(10^3^)^28/2^ = 10^42^	1.3 × 10^56^	94%
PPQ	(10^3^)^22/2^ = 10^33^	3.2 × 10^31^	80%
SP	(10^2^)^46/2^ = 10^46^	n/a	n/a
*Partner drugs (resistant):*			
PPQ_R_	(10^3^)^8/2^ = 10^12^	3.2 × 10^13^	55%
SP_R_	(10^2^)^9/2^ = 10^9^	n/a	n/a
*Artemisinins:*			
AR_q.d._	10^12^	4.8 × 10^12^	57.4%
AR_b.i.d_	10^20^	1.2 × 10^26^	91.8%
AS	10^12^	7.6 × 10^10^	43.2%
DHA	10^12^	5.0 × 10^10^	35.3%

The “simple” method uses Equation 1 for partner drugs, Equation 2 for artemisinins given once-daily over three days, and Equation 3 for artemether when given as six twice-daily doses. The PKPD method uses the approach outlined in Equation 4 and we include the partner drug monotherapy cure rates to quantify the degree of their drug sensitivity/resistance. The PRR_tot_ for the PKPD method are the median values shown in Fig. 1a (note that we cannot currently undertake a PKPD analysis of SP for reasons given in the Supplementary Information File). The PKPD method assumes wide, but continuous, ranges of values for the key PK and PD parameters (see Supplementary Information File) which results in the distributions of PRR_tot_ values on Fig. 1(a). This gives rise to an apparent discrepancy in this table i.e. that AQ has a lower therapeutic capacity (PRR_tot_) than PPQ but higher monotherapy cure rate. The reason is that PRR_tot_ given in the table is the median of the distribution simulated (Fig. 1(a)) whereas cure rates depend on the proportion of patients with low PRR_tot_. Patients given AQ in our parametrisation have relatively tightly clustered PRR_tot_ values which means the proportion of patients with a low PRR_tot_ is small (see 5^th^ centile values on Fig. 1(a)) so its failure rate is lower than for PPQ.

Abbreviations: AQ = amodiaquine, AR = artemether, AS = artesunate, DHA = dihydroartemisinin, LF = lumefantrine, MQ = mefloquine, n/a = not applicable, PKPD = pharmacokinetic-pharmacodynamic modelling, PPQ = piperaquine, SP = sulfadoxine-pyrimethamine;. Subscripts: b.i.d = twice daily dosing, R = resistance, q.d. = once daily dosing.

**Table 2:** The contribution of artemisinins to total ACT therapeutic capacity.

ACT	Simple method	PKPD method
*No resistance to partner drugs:*		
AQ + AS	1 × 10^−10.5^	2.1 × 10^−9^
LF + AR_b.i.d_	4 × 10^−17^	3.5 × 10^−10^
MQ + AS	1 × 10^−30^	5.4 × 10^−46^
PPQ + DHA	1 × 10^−21^	5.1 × 10^−22^
SP + AS	1 × 10^−34^	n/a
*Parasites resistant to partner drugs:*		
PPQ_R_ + DHA	1 × 10^0^	9.9 × 10^−5^
SP_R_ + AS	1 × 10^3^	n/a

This is quantified as the ratio of the artemisinin PRR_tot_ to partner drug PRR_tot_ using the values in Table 1. The contribution for the PKPD method are the median values shown in Fig. 1b.

Abbreviations: ACT = artemisinin combination therapy, AQ = amodiaquine, AR = artemether, AS = artesunate, auDKC = area under the drug kill curve, DHA = dihydroartemisinin, LF = lumefantrine, MQ = mefloquine, n/a = not applicable, PKPD = pharmacokinetic-pharmacodynamic modelling, PPQ = piperaquine, SP = sulfadoxine-pyrimethamine; Subscripts: b.i.d = twice daily dosing, R = resistance.

## References

[b1] World Health Organization. World Malaria Report 2015. *URL:* http://www.who.int/malaria/publications/world-malaria-report-2015/report/en/ (2016).

[b2] World Health Organization. *Emergency response to artemisinin resistance in the greater Mekong subregion. Regional Framework for action 2013–2015*. (World Health Organization, 2013).

[b3] Fairhurst R. M. & Dondorp A. M. Artemisinin-Resistant *Plasmodium falciparum* Malaria. Microbiol. Spectr. 4, EI10–0013. doi: 10.1128/microbiolspec.EI10-0013-2016 (2016). PMC4992992 27337450

[b4] Greenwood B. Treatment of malaria - A continuing challenge. N. Eng. J. Med. 371, 474–475 (2014). 10.1056/NEJMe1407026 25075840

[b5] Fairhurst R. M. *et al.* Artemisinin-resistant malaria: Research challenges, opportunities, and public health implications. Am. J. Trop. Med. Hyg. 87, 231–241 (2012). 22855752 10.4269/ajtmh.2012.12-0025 PMC3414557

[b6] International Artemisinin Study Group. Artesunate combinations for treatment of malaria: meta-analysis. *Lancet* 363, 9-17 (2004). 10.1016/s0140-6736(03)15162-8 14723987

[b7] Hastings I. M. How artemisinin-containing combination therapies slow the spread of antimalarial drug resistance. Trends in Parasitol. 27, 67–72 (2011). 10.1016/j.pt.2010.09.005 20971040

[b8] Hastings I. M. & Watkins W. M. Tolerance is the key to understanding antimalarial drug resistance. Trends in Parasitol. 22, 71–77 (2006). 10.1016/j.pt.2005.12.011 16406706

[b9] Hastings I. M., Watkins W. M. & White N. J. The evolution of drug resistant malaria; the role of drug elimination half-life. Phil. Trans. R. Soc. Lond. [B] 357, 505–519 (2002). 10.1098/rstb.2001.1036 PMC1692966 12028788

[b10] Kay K. & Hastings I. M. Measuring windows of selection for anti-malarial drug treatments. Malaria Journal 14, 1–10, doi: 10.1186/s12936-015-0810-4 (2015). 26228915 PMC4521485

[b11] Saunders D. L., Vanachayangkul P. & Lon C. Dihydroartemisinin–Piperaquine Failure in Cambodia. N. Eng J. Med. 371, 484–485, doi: 10.1056/NEJMc1403007 (2014). 25075853

[b12] Amaratunga C. *et al.* Dihydroartemisin-piperaquine resistance in *Plasmodium falciparum* malaria in Cambodia: a multisite prospective cohort study. Lancet Infect. Dis. 16, 357–365, doi: 10.1016/S1473-3099(15)00487-9 (2016). 26774243 PMC4792715

[b13] Phyo A. P. *et al.* Declining Efficacy of Artemisinin Combination Therapy Against *P. falciparum* Malaria on the Thai–Myanmar Border (2003–2013): The Role of Parasite Genetic Factors. Clin. Infect. Dis. doi: 10.1093/cid/ciw388 (2016). PMC4996140 27313266

[b14] Bhatt S. *et al.* The effect of malaria control on Plasmodium falciparum in Africa between 2000 and 2015. Nature 526, 207–211, doi: 10.1038/nature15535 (2015). 26375008 PMC4820050

[b15] Johnston G. L., Gething P. W., Hay S. I., Smith D. L. & Fidock D. A. Modeling Within-Host Effects of Drugs on *Plasmodium falciparum* Transmission and Prospects for Malaria Elimination. PLoS Comput Biol 10, doi: 10.1371/journal.pcbi.1003434 (2014). PMC3900379 24465196

[b16] Phyo A. P. *et al.* Emergence of artemisinin-resistant malaria on the western border of Thailand: A longitudinal study. Lancet 379, 1960–1966 (2012). 22484134 10.1016/S0140-6736(12)60484-X PMC3525980

[b17] Ashley E. A. *et al.* Spread of Artemisinin Resistance in *Plasmodium falciparum* Malaria. N. Engl. J. Med. 371, 411–423, doi: 10.1056/NEJMoa1314981 (2014). 25075834 PMC4143591

[b18] Stepniewska K. & White N. J. Pharmacokinetic determinants of the window of selection for antimalarial drug resistance. Antimicrob. Agents Chemother. 52, 1589–1596 (2008). 18299409 10.1128/AAC.00903-07 PMC2346628

[b19] Sisowath C. *et al.* *In vivo* selection of *Plasmodium falciparum* pfmdr 1 86N coding alleles by Artemether-Lumefantrine (Coartem). J. Infect. Dis. 191, 1014–1017 (2005). 15717281 10.1086/427997

[b20] Hastings I. M. & Ward S. A. Coartem in Africa- the beginning of the end? J. Infect. Dis. 192, 1303–1304 (2005). 16136476 10.1086/432554

[b21] Kay K., Hodel E. M. & Hastings I. M. Altering antimalarial drug regimens may dramatically enhance and restore drug effectiveness. Antimicrob. Agents Chemother. 59, 6419–6427, doi: 10.1128/aac.00482-15 (2015). 26239993 PMC4576027

[b22] Ndour P. A. *et al.* *Plasmodium falciparum* clearance is rapid and pitting independent in immune Malian children treated with artesunate for malaria. J Infect Dis 211, 290–297, doi: 10.1093/infdis/jiu427 (2015). 25183768 PMC4334830

[b23] Lopera-Mesa T. M. *et al.* *Plasmodium falciparum* clearance rates in response to artesunate in Malian children with malaria: Effect of acquired immunity. J. Infect. Dis. 207, 1655–1663 (2013). 23448727 10.1093/infdis/jit082 PMC3636783

[b24] Hastings I. M., Kay K. & Hodel E. M. How robust are malaria parasite clearance rates as indicators of drug effectiveness and resistance? Antimicrob. Agents Chemother. 59, 6428–6436, doi: 10.1128/aac.00481-15 (2015). 26239987 PMC4576129

[b25] Ferreira P. E., Culleton R., Gil J. P. & Meshnick S. R. Artemisinin resistance in *Plasmodium falciparum*: what is it really? Trends Parasitol 29, 318–320, 10.1016/j.pt.2013.05.002 (2013). 23768531

[b26] Meshnick S. Perspective: Artemisinin-resistant malaria and the wolf. Am. J. Trop. Med Hyg. 87, 783–784 (2012). 23136171 10.4269/ajtmh.2012.12-0388 PMC3516250

[b27] Krishna S. & Kremsner P. G. Antidogmatic approaches to artemisinin resistance: reappraisal as treatment failure with artemisinin combination therapy. Trends Parasitol. 29, 313–317 (2013). 23623760 10.1016/j.pt.2013.04.001

[b28] Ursing J. *et al.* Similar efficacy and tolerability of double-dose chloroquine and artemether-lumefantrine for treatment of Plasmodium falciparum infection in Guinea-Bissau: a randomized trial. J. Infect. Dis. 203, 109 (2011). 21148503 10.1093/infdis/jiq001 PMC3086436

[b29] White N. J. Pharmacokinetic and pharmacodynamic considerations in antimalarial dose optimization. Antimicrob. Agents Chemother. 57, 5792–5807, doi: 10.1128/aac.00287-13 (2013). 24002099 PMC3837842

[b30] Spring M. D. *et al.* Dihydroartemisinin-piperaquine failure associated with a triple mutant including kelch13 C580Y in Cambodia: an observational cohort study. Lancet Infect. Dis. 15, 683–691, doi: 10.1016/S1473-3099(15)70049-6 (2015). 25877962

[b31] Shanks G. D., Edstein M. D. & Jacobus D. Evolution from double to triple-antimalarial drug combinations. Trans. R. Soc. Trop. Med. Hyg., doi: 10.1093/trstmh/tru199 (2014). 25549631

[b32] *A Study by the Tracking Resistance to Artemisinin Collaboration (TRAC*) (*TRACII*), https://clinicaltrials.gov/ct2/show/NCT02453308 (2015).

[b33] malERA. A Research Agenda for Malaria Eradication: Modeling. *PLoS Med* 8, e1000403, doi: 10.1371/journal.pmed.1000403 (2011). 10.1371/journal.pmed.1000403 PMC3026697 21283605

[b34] Slater H. C., Griffin J. T., Ghani A. C. & Okell L. C. Assessing the potential impact of artemisinin and partner drug resistance in sub-Saharan Africa. Malaria J. 15, 1–11, doi: 10.1186/s12936-015-1075-7 (2016). PMC4704433 26739092

[b35] White N. J. The assessment of antimalarial drug efficacy. Trends Parasitol. 18, 458–464 (2002). 12377597 10.1016/s1471-4922(02)02373-5

[b36] White N. J. Assessment of the pharmacodynamic properties of antimalarial drugs *in vivo*. Antimicrob. Agents Chemother. 41, 1413–1422 (1997). 9210658 10.1128/aac.41.7.1413 PMC163932

[b37] Ross R. Observations On The Principle Of Repeated Medication For Curing Infections. The Brit. Med. J.l 2, 1–4, doi: 10.2307/20427895 (1921). PMC2339430 20770357

[b38] Kay K. & Hastings I. M. Improving pharmacokinetic-pharmacodynamic modeling to investigate anti-infective chemotherapy with application to the current generation of antimalarial drugs. PLoS Comput Biol 9, e1003151, doi: 10.1371/journal.pcbi.1003151 (2013). 23874190 PMC3715401

[b39] Czock D. & Keller F. Mechanism-based pharmacokinetic-pharmacodynamic modeling of antimicrobial drug effects. J. Pharmacokinet. Pharmacodyn. 34, 727–751 (2007). 17906920 10.1007/s10928-007-9069-x

[b40] Hoshen M. B., Stein W. D. & Ginsburg H. Modelling the chloroquine chemotherapy of falciparum malaria: the value of spacing a split dose. Parasitology 116, 407–416 (1998). 9614323 10.1017/s0031182098002480

[b41] Austin D. J., White N. J. & Anderson R. M. The dynamics of drug action on the within-host population growth of infectious agents: melding pharmacokinetics with pathogen population dynamics. J. Theor. Biol. 194, 313–339 (1998). 9778442 10.1006/jtbi.1997.0438

[b42] Hoshen M. B., Heinrich R., Stein W. D. & Ginsburg H. Mathematical modelling of the within-host dynamics of *Plasmodium falciparum*. Parasitology 121, 227–235 (2000). 11085243 10.1017/s0031182099006368

[b43] Hoshen M. B., Na-Bangchang K., Stein W. D. & Ginsburg H. Mathematical modelling of the chemotherapy of Plasmodium falciparum malaria with artesunate: postulation of ‘dormancy’, a partial cytostatic effect of the drug, and its implication for treatment regimens. Parasitology 121, 237–246 (2000). 11085244 10.1017/s0031182099006332

[b44] Hoshen M. B., Stein W. D. & Ginsburg H. Mathematical modelling of malaria chemotherapy: combining artesunate and mefloquine. Parasitology 124, 9–15 (2002). 11811806 10.1017/s0031182001008952

[b45] Hoshen M. B., Stein W. D. & Ginsburg H. D. Pharmacokinetic-pharmacodynamic modelling of the antimalarial activity of mefloquine. Parasitology 123, 337–346 (2001). 11676365 10.1017/s003118200100854x

[b46] Simpson J. A. *et al.* Mefloquine pharmacokinetic-pharmacodynamic models: Implications for dosing and resistance. Antimicrob. Agents Chemother. 44, 3414–3424 (2000). 11083649 10.1128/aac.44.12.3414-3424.2000 PMC90214

[b47] Zaloumis S. *et al.* Assessing the utility of an anti-malarial pharmacokinetic-pharmacodynamic model for aiding drug clinical development. Malaria J.l 11, 303 (2012). 10.1186/1475-2875-11-303 PMC3546862 22931058

[b48] Hastings I. & Hodel E. M. Pharmacological considerations in the design of anti-malarial drug combination therapies - is matching half-lives enough? Malaria J. 13, 62 (2014). 10.1186/1475-2875-13-62 PMC3975950 24552440

[b49] Hodel E., Kay K., Hayes D., Terlouw D. & Hastings I. Optimizing the programmatic deployment of the anti-malarials artemether-lumefantrine and dihydroartemisinin-piperaquine using pharmacological modelling. Malaria J. 13, 138 (2014). 10.1186/1475-2875-13-138 PMC4036747 24708571

[b50] Hodel E. M., Kay K. & Hastings I. M. Incorporating stage specificity into pharmacological modelling of antimalarial drug treatment. Antimicrob. Agents Chemother. in press, doi: 10.1128/AAC.01172-15 (2016). PMC4862506 26902760

[b51] Kay K., Hodel E. M. & Hastings I. M. Improving the role and contribution of pharmacokinetic analyses in antimalarial drug clinical trials. Antimicrob. Agents Chemother. 58, doi: 10.1128/aac.02777-14 (2014). PMC4187976 24982091

[b52] Winter K. & Hastings I. M. Development, evaluation and application of an *in silico* model for antimalarial drug treatment and failure. Antimicrob. Agents Chemother. 55, 3380–3392 (2011). 21537019 10.1128/AAC.01712-10 PMC3122393

