# Supplementary material for: Quantifying the pharmacology of antimalarial drug combination therapy
Source: Sci Rep. 2016 Sep 8;6:32762. doi: 10.1038/srep32762 (PMC5036534; doi:10.1038/srep32762)

Supplementary Information File.

Quantifying the pharmacology of antimalarial drug combination therapy.

Ian M. Hastings, Eva Maria Hodel, Katherine Kay

The following abbreviations are used for drugs in this Supplementary Information File.
Partner drugs: AQ = amodiaquine, LF = lumefantrine, MQ = mefloquine, PPQ = piperaquine, SP = sulfadoxine-pyrimethamine.
Artemisnins: AR = artemether, AS = artesunate, DHA = dihydroartemisinin.

In this work, we focus on quantifying the therapeutic capacities of drugs prior to resistance evolving against them; the two exceptions are PPQ and SP. Resistance has already arisen to both PPQ and SP, and so we have data upon which to base these metrics (see below). In contrast, data for AQ and MQ are very poor, and there is no data currently available for LF as there is little evidence of resistance arising to the latter drug. We wish to avoid making arbitrary guestimates of the likely impact if/when resistance arises to AQ, LF, or MQ so have not done so. We also do not explicitly investigate artemisinin resistance for a similar reason. Artemisinin “resistance” is actually only partial resistance [1]: the loss of drug sensitivity is restricted to the first few hours after parasites invade red blood cells and parasites are believed to remain sensitive to artemisinins in the other susceptible stages of their 48-hour developmental cycle. We could easily decrease artemisinin killing rate to reflect this partial resistance (for example, cutting PRR_{48} from it consensus value of 10^4 for sensitivity parasites down to 10^3 for partially resistant forms) but wish to avoid taking such an arbitrary approach to artemisinin resistance. To date, it appears that no-one has quantified the impact of this partial resistance on overall killing and we are reluctant to attempt it in this paper. Criticism of our choice of values could detract from the basic message of this paper, i.e. that artemisinins, even in the absence of resistance, have extremely small therapeutic capacities compared to their partner drugs. Hence key conclusions are conservative i.e. the therapeutic capacity of artemisinins compared to parent drugs (Table 2 and Fig. 1b) will become even smaller as artemisinin resistance spreads.

(1) Calibrations for analyses using the simple method

Calibrations of artemisinins are as described in the main text (i.e. Equations 2 and 3). Calibrating for partner drugs requires an estimate of their persistence at active concentrations post-treatment (i.e. d in Equation 1 of the main text). In fact, it is their duration post-treatment when they are killing at maximum rates (i.e. at concentrations that generate their observed PRR_{48}) that needs to be estimated. The drug concentrations of long half-live partner drugs decline gradually but, as discussed previously (Fig. 3 to 5 of reference [2]) the non-linearity of Michaelis-Menton mechanics means that their kill rates drop much more suddenly than their concentrations. Ignoring drug killing during the few days as they decline from maximal to zero will underestimate partner drug killing but not by a large amount (and note that the more sophisticated PKPD method, described below, does incorporate killing during this transitional phase). We therefore use the length of time post-treatment that drugs prevent new infections as an estimate of persistence post-treatment. New infections emerge from the liver as a cohort of ~10^5 parasites but do not become patent (i.e. identifiable under the microscope) until they

reach 10^8 parasites so this lag period needs to be incorporated. Rates of malaria growth *in vivo* vary substantially (discussed in supplemental material of reference [3]) but we assume here that parasites multiply 10-fold every 48 hour parasite cycle as in previous analyses by us (e.g. reference [3,4]) and others (e.g. reference [5]), resulting in a lag period of 6 days to allow new infections to grow to patency. We therefore estimate the time of effective drug persistence post-treatment as the durations of its protective effect post-treatment (obtained from the literature as described below) minus six days.

Calibration also required an estimate of PRR_{48}. Most estimates derive from a common source (Table 1 of reference [6]) which reviewed previous estimates and noted that immunity also affected PRR_{48}. We previously assumed a PRR_{48} of 10^4 for artemisinins and 10^3 for the partner drugs (except sulfadoxine-pyrimethamine, SP); our analyses were consistent with field data and we continue to use these PRR values here. White (Table 1 of reference [6]) estimates PRR_{48} for SP as between 10 and 10^3, we use a value of 10^2.

Amodiaquine (AQ). We are unaware of re-infection studies that would enable us to estimate the duration of prophylaxis post-treatment. We therefore use some recent modelling results to determine the time until a typical patient's desethyl-amodiaquine (DEAQ), the active metabolite of AQ, levels fall below twice the half maximal inhibitory concentration (IC_{50}), giving a duration of protection of around 12 days (Table 4.1 and Appendix 2 of reference [7]).

Lumefantrine (LF). Sisowath and colleagues (reference [8], see also reference [9]) presented re-infection rates following treatment with artemether-lumefantrine which enabled us to estimate the protective period of sensitive parasites as 40 days [10], giving estimated persistence at active concentrations as 34 days (note that in our earlier interpretation [10] we assumed 10 days for new infections to become patent compared to the 6 days used here and justified above). Note that their data also report re-infections times for parasites carrying less drug-sensitive alleles but we do not regard them as clinically resistant because their level of reduced sensitivity was insufficient for therapeutic failures to occur. In contrast, a recent comparison of PPQ and LF across six sites in Africa suggested re-infection following LF started to rise around 15 days post treatment [11] although this may reflect an increase in LF tolerance [12] that may have occurred since the original Sisowath *et al*. study. We therefore take the mid-point value of the studies i.e. (34 + 15) / 2 = 24.5 days

Mefloquine (MQ). A recent Cochrane review reported that both PPQ and MQ had "very long half-lives and no consistent benefit in preventing new infections has been seen over 63 days follow up" [13]. Our previous pharmacological analyses [3] and sporadic reports in the literature (e.g. reference [14]) suggest that MQ may persistent slightly longer so we estimate it to last for 28 days in MQ (as opposed to 22 days for PPQ; see below)

Sulphadoxine-pyrimethamine (SP). Watkins and Mosobo[15] report duration of protection post-treatment is 52 days against fully sensitive forms, and 15 days against resistance parasites; these figures were also used later in the calculations in reference [16]. This gives duration of persistence as 46 and 9 days for sensitive and resistant forms respectively.

Piperaquine (PPQ). Calibration for this drug is challenging as PPQ is highly sensitive to levels of resistance (defined by increasing IC_{50} values) in the parasite population. Depending on the study, PPQ either shows a two- or three-compartment pharmacokinetic (PK) disposition, which means concentration in the blood initially falls rapidly after dosing, followed by a long terminal elimination phase. Sensitive parasites are killed by drug

concentrations in the terminal elimination phase but as resistance increases, the parasites become insensitive to these persisting low concentrations and the period of killing post-treatment falls dramatically (see Fig. 2 of reference [17]). This is supported by clinical data [18] where DHA + PPQ failure rates rose dramatically after just a 3-fold increase in PPQ IC_{50} and that times until observed first new infection fell from 28 days to 14 days. The first figure is consistent with a comparison across several sites which reported re-infection rates started to rise only after around 30 days [11]. We therefore estimate periods of protection post treatment as 22 days and 8 days for sensitive and resistant parasites respectively.

(2) Calibrations for analyses using pharmacokinetic/pharmacodynamics (PKPD) modelling.

Different methods are required to obtain $f(D)$ in Equation 4 of the main text because drugs differ in how they are distributed (one-, two-, or three-compartment drug disposition) and/or whether they are converted to active metabolites. The methods used here are already in the literature and here we provide details of the PK model used, the supporting references, and the means and variances of the PK and PD parameters. We cite our previous studies at numerous points; readers can use these publications to gain access to the relevant literature on antimalarial PK and PD estimation upon which we based our parameterisations, and see how we incorporated these parameter values (for example, how we dealt with the extensive variance surrounding some key estimates). Our work has developed, and become more nuanced, over a period of time. For example, we initially modelled LF assuming it has a one-compartment PK disposition although it should arguably be two-compartmental. Consequently, we faced the choice of re-modelling LF or using the model and estimates we had already published [19]. We chose the latter course as it avoids raising any suspicions that we may be re-simulating results to better fit our hypothesis, and because readers can be assured that it has already been through a peer-review process; essentially, even if the methodology was an over-simplification, the results did correlate well with clinical and field observations. The reference to "instantaneous" absorption mentioned below refers to our (and other authors e.g. [20,21]) assumption that the drug instantly enters the blood stream after the patient swallows the treatment (i.e. the "bolus"). First-order absorption across the gut wall is more realistic, but the assumption of instantaneous absorption makes the PK calculus simple and it is likely that the time difference between these approaches, which in reality will be a few hours, is negligible in drugs whose half-lives may be days. We must emphasise that these assumptions were only applied to the partner drugs and the artemisinin calculations were appropriate i.e. we did include first-order absorption across the gut because it does constitute an important part of the timeframe for artemisinin action, and we did model artemisinins using the appropriate one-compartment PK model (see below for specific details).

Artesunate (AS). First order absorption, one-compartment PK disposition with conversion to its active metabolite DHA: dosage, methods and calibration as described previously [4].

Artemether (AR). First order absorption, one-compartment PK disposition with conversion to DHA: dosage, methods and calibration as described previously [4,22].

Dihydroartemisinin (DHA). First order absorption, one-compartment PK disposition and linear elimination: dosage, methods and calibration as described previously [4].

Amodiaquine (AQ). First order absorption and conversion into its active metabolite desethyl-amodiaquine (DEAQ). Both AQ and DEAQ have anti-malarial activity and both are distributed in to two-PK compartments and eliminated[23] . We therefore used the PKPD method of [24] who tracked both AQ and DEAQ separately but simultaneously. Calibration was as described previously [7] and was designed to match an efficacy of the AS-AQ combination of 85%; this gave an efficacy of AQ monotherapy as 35%. This was obtained using a PRR of 10^2 for AQ which is at the lower end of the estimated PRR (ranging from 10^2 to 10^4 in reference [6]). We therefore reverted to using a PRR of 10^3 which gave an AQ monotherapy cure rate of 86.5%.

Lumefantrine (LF). Instantaneous absorption, one-compartment PK disposition and linear elimination: dosage, methods and calibration as described previously [3].

Mefloquine (MQ). Instantaneous absorption, one-compartment PK disposition and linear elimination: dosage, methods and calibration as described previously [3].

Piperaquine (PPQ). First-order absorption, two-compartment PK disposition and linear elimination: dosage, methods and calibration as described previously [22].

Sulphadoxine-pyrimethamine (SP). We attempted a pharmacological analysis of this drug combination using the PD data presented as an isobologram by Gatton and colleagues [25]. Our approach was successful for its specific application to intermittent prevent treatment in pregnancy (IPTp) [26] but is not suitable in the current context. The two drugs exhibit synergy which brings huge complexity into the modelling; few factors in pharmacology are as contentious (and often contradictory) as synergy, see Chou [27] for a very extensive discussion. It was therefore extremely difficult to incorporate parasite variation in drug sensitivity to the combination. Given this uncertainly in the PD element, we decided not to include an attempted PKPD analysis of SP.

(3) Methodological notes

The PKPD method. The PPR_{tot} was obtained for each drug by finding the minimum number of parasites post treatment, and dividing the initial parasite number P_0 by this minimum number (the value of P_0 should not affect the value of PPR_{tot} but, for consistency with previous analyses[4], we selected P_0 from a uniform distribution between 10^{10} and 10^{12}). We simulated 1,000 patients with variable pharmacological parameters to incorporate the patients' inter-individual variability and the natural variability between parasites; calibration details can be found above. The median PPR_{tot} for each drug is given on Table 1 of the main text and the population variability shown in Fig. 1a. The PRR_{tot} ratio was used to determine the contribution of artemisinin killing to the total ACT killing in 1,000 patients. The ratio was found by randomly selecting one previously generated PPR_{tot} value from the artemisinin species and one PPR_{tot} value from the corresponding partner drug in the ACT. This allowed us to incorporate population variability, assuming (reasonably in our opinion) that artemisinin and partner drugs PK are independent, as are parasites' sensitivity to the two drugs. The median PRR_{tot} ratio is given on Table 2 of the main text and the population variability shown in Fig. 1b.

The "auDKC" approach. Consideration of Equation 4 in the main text shows that parasite growth rate, *a*, also affects PRR_{tot}. The integral in that Equation 4 is the area under the drug-kill curve (auDKC) whose magnitude is independent of *a* and is therefore an alternative metric of overall drug killing. It was used previously by us [2,22] so we also calculated the auDKC for the main ACT drugs for consistency with this earlier work, and quantified the contribution of artemisinins to overall ACT killing as the ratio of the auDKC of artemisinin-to-partner. The results are given in Fig. S1 and, as expected, are qualitatively consistent with the PRR_{tot} approach used in the main text (Fig. 1b of main text) in that artemisinin contribution to overall killing is low.

Reference List

1 Fairhurst, R. M. & Dondorp, A. M. Artemisinin-Resistant *Plasmodium falciparum* Malaria. *Microbiol. Spectr.* **4**, EI10-0013 doi:doi:10.1128/microbiolspec.EI10-0013-2016 (2016).

2 Hastings, I. & Hodel, E. M. Pharmacological considerations in the design of anti-malarial drug combination therapies - is matching half-lives enough? *Malaria J* **13**, 62 (2014).

3 Winter, K. & Hastings, I. M. Development, evaluation and application of an *in silico* model for antimalarial drug treatment and failure. *Antimicrob. Agents Chemother.* **55**, 3380-3392 (2011).

4 Kay, K. & Hastings, I. M. Improving pharmacokinetic-pharmacodynamic modeling to investigate anti-infective chemotherapy with application to the current generation of antimalarial drugs. *PLoS Comput. Biol.* **9**, e1003151, doi:10.1371/journal.pcbi.1003151 (2013).

5 Simpson, J. A. *et al.* Mefloquine pharmacokinetic-pharmacodynamic models: Implications for dosing and resistance. *Antimicrob. Agents Chemother.* **44**, 3414-3424 (2000).

6 White, N. J. Assessment of the pharmacodynamic properties of antimalarial drugs in vivo. *Antimicrob. Agents Chemother.* **41**, 1413-1422 (1997).

7 Hong, K. B. *Pharmacological modelling of the efficacy and safety of fixed-dose versus non-fixed dose combinations of the antimlarial drug artesunate-amodiaquine* MSc thesis, Liverpool School of Tropical Medicine, (2014).

8 Sisowath, C. *et al.* In vivo selection of *Plasmodium falciparum* pfmdr 1 86N coding alleles by Artemether-Lumefantrine (Coartem). *J. Infect. Dis.* **191** 1014-1017 (2005).

9 Sisowath, C. *et al.* The role of pfmdr1 in *Plasmodium falciparum* tolerance to artemether-lumefantrine in Africa. *Trop. Med. Int. Health* **12**, 736-742 (2007).

10 Hastings, I. M. & Ward, S. A. Coartem in Africa- the beginning of the end? *J. Infect. Dis.* **192**, 1303-1304 (2005).

11 Okell, L. C. *et al.* Contrasting benefits of different artemisinin combination therapies as first-line malaria treatments using model-based cost-effectiveness analysis. *Nat. Commun.* **5**, doi:10.1038/ncomms6606 (2014).

12 Plucinski, M. M. *et al.* Efficacy of Artemether-Lumefantrine and Dihydroartemisinin-Piperaquine for Treatment of Uncomplicated Malaria in Children in Zaire and Uíge Provinces, Angola. *Antimicrob. Agents Chemother.* **59**, 437-443, doi:10.1128/aac.04181-14 (2015).

13 Zani, B., Gathu, M., Donegan, S., Olliaro, P. L. & Sinclair, D. Dihydroartemisinin-piperaquine for treating uncomplicated *Plasmodium falciparum* malaria. *Cochrane Database Syst Rev*, 1-160, doi:10.1002/14651858.CD010927 (2014).

14 Grande, T. *et al.* A Randomised Controlled Trial to Assess the Efficacy of Dihydroartemisinin-Piperaquine for the Treatment of Uncomplicated Falciparum Malaria in Peru. *PLoS ONE* **2**, e1101, doi:10.1371/journal.pone.0001101 (2007).

15 Watkins, W. M. & Mosobo, M. Treatment of Plasmodium falciparum malaria with pyrimethamine-sulfadoxine: selective pressure for resistance is a function of long elimination half-life. *Trans. R. Soc. Trop. Med. Hyg.* **87**, doi:10.1016/0035-9203(93)90431-o (1993).

16 Hastings, I. M., Watkins, W. M. & White, N. J. The evolution of drug resistant malaria; the role of drug elimination half-life. *Phil.l Trans. Roy. Soc. Lond. [B]* **357**, 505-519 (2002).

17 Kay, K. & Hastings, I. M. Measuring windows of selection for anti-malarial drug treatments. *Malaria J* **14**, 1-10, doi:10.1186/s12936-015-0810-4 (2015).

18 Saunders, D. L., Vanachayangkul, P. & Lon, C. Dihydroartemisinin–Piperaquine Failure in Cambodia. *N. Engl. J. Med.* **371**, 484-485, doi:doi:10.1056/NEJMc1403007 (2014).

19 Hodel, E., Kay, K., Hayes, D., Terlouw, D. & Hastings, I. Optimizing the programmatic deployment of the anti-malarials artemether-lumefantrine and dihydroartemisinin-piperaquine using pharmacological modelling. *Malaria J* **13**, 138 (2014).

20 Hoshen, M. B., Stein, W. D. & Ginsburg, H. Modelling the chloroquine chemotherapy of falciparum malaria: the value of spacing a split dose. *Parasitology* **116**, 407-416 (1998).
21 Simpson, J. A. *et al.* Mefloquine pharmacokinetic-pharmacodynamic models: implications for dosing and resistance. *Antimicrob. Agents Chemother.* **44**, doi:10.1128/aac.44.12.3414-3424.2000 (2000).
22 Kay, K., Hodel, E. M. & Hastings, I. M. Altering antimalarial drug regimens may dramatically enhance and restore drug effectiveness. *Antimicrob. Agents Chemother.* **59**, 6419-6427, doi:10.1128/aac.00482-15 (2015).
23 Hietala, S. *et al.* Population pharmacokinetics of amodiaquine and desethylamodiaquine in pediatric patients with uncomplicated falciparum malaria. *J Pharmacokinet Pharmacodyn* **34**, 669 - 686 (2007).
24 Stepniewska, K. *et al.* Population pharmacokinetics of artesunate and amodiaquine in African children. *Malaria J* **8**, 200 (2009).
25 Gatton, M. L., Martin, L. B. & Cheng, Q. Evolution of resistance to sulfadoxine-pyrimethamine in Plasmodium falciparum. *Antimicrob. Agents Chemother.* **48**, 2116-2123 (2004).
26 Htay, M. N. N. *Pharamcological modelling of intermittent preventative treatment in pregnancy with sulpadoxine-pyrimethamine* MSc thesis, Liverpool School of Tropical Medicine, (2014).
27 Chou, T. C. Theoretical basis, experimental design, and computerized simulation of synergism and antagonism in drug combination studies. *Pharmacol. Rev.* **58**, 621-681 (2006).

Table S1. **The contribution of artemisinins to total ACT therapeutic capacity killing using the auDKC approach.** This is quantified as the ratio of the artemisinin PRR_{tot} to partner drug PRR_{tot} using the median values shown on Fig. S1.

**ACT**	**Artemisinin contribution to killing**
No resistance to partner drugs:	
**AQ + AS**	5.4×10^{-70}
**LF + $AR_{b.i.d}$**	2.6×10^{-28}
**MQ + AS**	4.7×10^{-107}
**PPQ + DHA**	6.3×10^{-49}
**SP + AS**	n/a
Parasites resistant to partner drugs:	
**PPQ_R + DHA**	1.3×10^{-16}
**SP_R+ AS**	n/a

Abbreviations: ACT = artemisinin combination therapy, AQ = amodiaquine, AR = artemether, AS = artesunate, auDKC = area under the drug kill curve, DHA = dihydroartemisinin, LF = lumefantrine, MQ = mefloquine, n/a = not applicable, PPQ = piperaquine, SP = sulfadoxine-pyrimethamine; Subscripts: b.i.d = twice daily dosing, R = resistance.

Figure S1. The contribution of artemisinin to overall ACT therapeutic capacity. This is quantified as the ratio of the area under their drug kill curve (auDKC) of artemisinin: partner drugs. Note that in all plots the upper "whisker" of the boxplot lies immediately above the box and is difficult to distinguish. We also identify the 5th and 95th centile of the data by horizontal red lines. The structure of this plot is as explained on the Caption to Fig. 1 in the main text.

Abbreviations: ACT = artemisinin combination therapy, AQ = amodiaquine, AR = artemether, AS = artesunate, DHA = dihydroartemisinin, LF = lumefantrine, MQ = mefloquine, PPQ = piperaquine, SP = sulfadoxine-pyrimethamine; Subscripts: R = resistance.

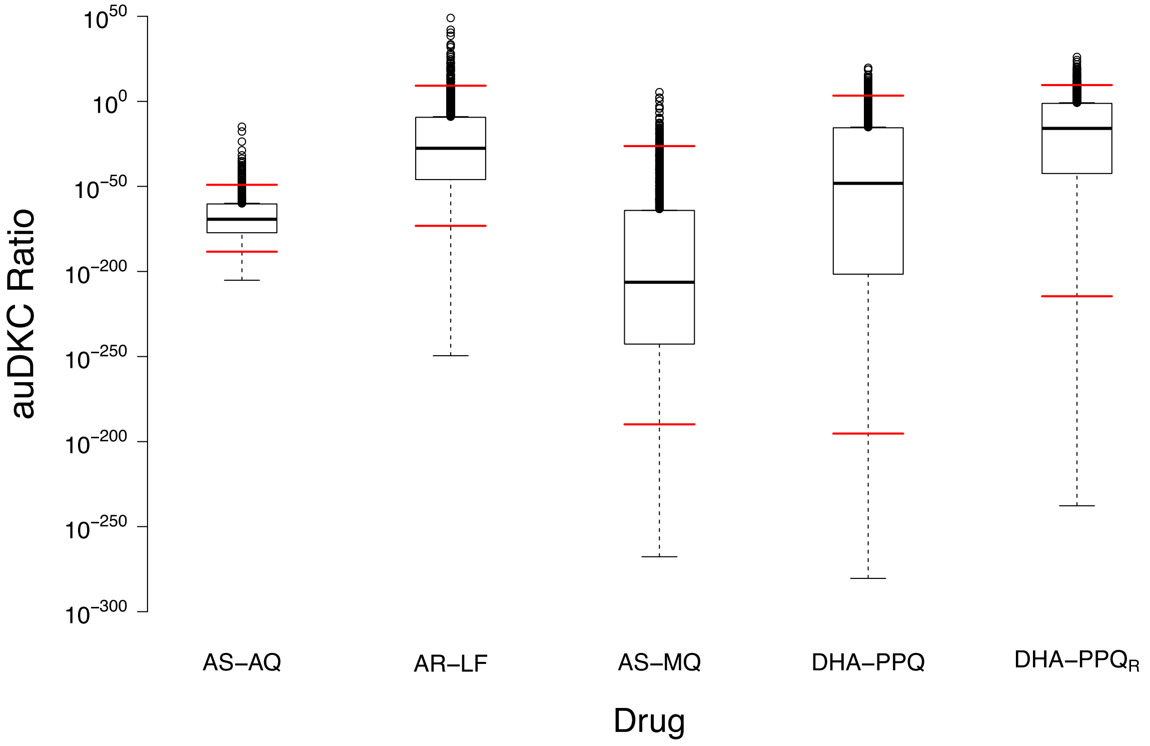

Supplement: Supplementary Information [file srep32762-s1.pdf]
